# Channelling protons out of the heart

**DOI:** 10.1113/JP283250

**Published:** 2022-05-11

**Authors:** Pawel Swietach, Sanda Despa

**Affiliations:** ^1^ Department of Physiology Anatomy & Genetics Oxford UK; ^2^ Department of Pharmacology and Nutritional Sciences University of Kentucky Lexington Kentucky USA

**Keywords:** acid‐base balance, cardiomyocyte, mitochondria, voltage‐gated channel

Acid‐base carriers were among the first membrane transport processes to be investigated in the 1970s. Since the advent of methods for isolating cells, many studies of pH regulation used cardiac myocytes, ostensibly because of the clinical relevance of acidosis in ischaemia. Ma et al. (2022) present possibly the biggest paradigm shift in the field in 20 years by showing evidence for a role of HVCN1 voltage‐gated H^+^ channels in expunging acid from cells, at least in canine myocytes. Unlike Na^+^/H^+^ exchanger‐1 (NHE1), the heart's most celebrated pH regulator, HVCN1 is electrogenic and not stoichiometrically coupled to Na^+^ uptake. The authors argue that this novel H^+^ extrusion pathway would be cardioprotective, as it avoids disrupting calcium signalling secondary to Na^+^ overload – an issue with Na^+^‐dependent acid‐extruders. The authors undertook extensive electrophysiological studies to verify their discovery. The fluxes attributed to HVCN1 are truly large; for example, dialysing cells with an acidic internal solution (pHi = 6.5) that normally triggers NHE1 activity in the order of tens of mm/min evoked an outward current of ∼1 pA/pF at 0 mV under near‐physiological extracellular pH. This can be converted to a H^+^ flux by considering the Faraday constant (96,500 A s/mol) and a representative ratio of membrane capacitance to cytosolic volume (Satoh et al., 1996) in mammalian myocytes (9 pF/pl). The calculated flux is 6 mmol/(l cytosol)/min, i.e. comparable to that produced by NHE1, bringing HVCN1 to the forefront of pH regulators. It remains unclear why previous studies, using the classical ammonium prepulse method, found no evidence for HVCN1‐dependent flux (e.g. complete block of pH recovery when NHE was inactivated in CO_2_/HCO_3_^−^‐free superfusates), but the present findings mandate a re‐evaluation of pH control in the heart. A role for HVCN1 may be important in the context of ischaemia and reperfusion, as well as cardiac hypertrophy.

Evidence points to the current being H^+^‐selective, Nernstian and highly temperature sensitive: the hallmarks of HVCN1 channels. Pharmacological responses were also consistent with this type of channel. Possibly the most compelling test for the homeostatic significance of a novel transport mechanism is to demonstrate that its blockade evokes a meaningful change to the intracellular concentration of the carried ions. Under pharmacological NHE1 blockade, HVCN1 inhibition produced a rapid and profound acidification, interpreted to indicate considerable H^+^ traffic at resting pHi in paced cells. pHi decreased by ∼1 unit in 5 min, equivalent to an acidifying flux of ∼8 mmol l^−1^ min^−1^, if we take an average intrinsic buffering capacity over this pH range to be ∼40 mmol/(l cytosol)/pH (Leem et al., 1999). The source of this acid is unclear, but metabolism is one of the few processes capable of supplying such a sustained magnitude of acid. In well‐perfused myocytes, glycolytic lactic acid production is unlikely because oxygen supply is ample. The other major metabolic source of acid is mitochondrial CO_2_ production, which the authors postulate supplies the H^+^ ions that are ultimately extruded by HVCN1 channels.

The magnitude of CO_2_ production in the heart is well known. Myocardial O_2_ consumption is 8−13 ml/100 g of tissue/min (Hoffman & Buckberg, 2014). Taking Avogadro's law, the heart's respiratory quotient (CO_2_ production/O_2_ consumption) of 0.8, and that 100 g of myocardial tissue contains 69 ml of water (Vinnakota & Bassingthwaighte, 2004), the resting CO_2_ production rate is ∼6 mmol l^−1^ min^−1^. Since the electrically paced isolated myocytes under study were not mechanically loaded, their respiratory rate may be lower than this whole‐tissue estimate. Nonetheless, the flux of H^+^ ions carried by HVCN1 channels is similar to mitochondrial CO_2_ production. This is significant, because it would indicate that virtually all CO_2_ production is trafficked across the surface membrane as H^+^ and HCO_3_^−^ ions, rather than as CO_2_ gas. This is a striking observation because it represents a paradigm shift in our thinking of CO_2_ handling. So far, it was firmly believed that essentially all mitochondrially produced CO_2_ crosses the membrane as CO_2_ gas. Even though CO_2_ is hydrated in the cytoplasm by carbonic anhydrase enzymes, there will always remain a residual concentration of the gaseous form. According to the canonical model, mitochondrial CO_2_ is vented across membranes in its gaseous form, rather than as its dissociated ions, as these are considered to be several orders of magnitude less permeable than uncharged molecules. The new proposal, which states that HVCN1 current (alongside a high‐capacity HCO_3_^−^ permeability) carries the bulk of mitochondrial CO_2_ output, implies that the membrane must have restricted capacity to conduct CO_2_ gas, despite a favourable concentration gradient. In other words, if cardiomyocytes rely on HVCN1 channels to remove CO_2_, their gas permeability is much lower than previously measured (Arias‐Hidalgo et al., 2017). If HVCN1 channels are essential to overcome previously unrecognized gas permeability limitations, the findings are of major importance to our understanding of membrane biology. Moreover, to vent CO_2_ as its hydration products, it is essential to match H^+^ current with HCO_3_^−^; the identity of the latter process remains to be confirmed, with a possible role for Cl^−^/HCO_3_^−^ exchangers.

The discovery of HVCN1 current in canine myocytes is an important contribution to the field. Its implications on our understanding of CO_2_ permeability, as well as parallel HCO_3_^−^ conductances, are considerable and these need to be re‐evaluated. Further studies will certainly look into the role of HVCN1 channels in myocytes of other species, including humans and their role in heart diseases.

## Additional information

### Competing interests

None declared.

### Author contributions

Both authors have read and approved the final version of the manuscript submitted for publication. All persons designated as authors qualify for authorship, and all those who qualify for authorship are listed.

### Funding

British Heart Foundation (BHF): Pawel Swietach, RG/15/9/31534.

## Supporting information


Peer Review History

## References

[tjp15085-bib-0001] Arias‐Hidalgo, M. , Al‐Samir, S. , Weber, N. , Geers‐Knorr, C. , Gros, G. , & Endeward, V. (2017). CO_2_ permeability and carbonic anhydrase activity of rat cardiomyocytes. Acta Physiologica (Oxford, England), 221(2), 115–128. 10.1111/apha.12887 28429509

[tjp15085-bib-0002] Hoffman, J. I. , & Buckberg, G. D. (2014). The myocardial oxygen supply:demand index revisited. Journal of the American Heart Association, 3(1), e000285. 24449802 10.1161/JAHA.113.000285 PMC3959699

[tjp15085-bib-0003] Leem, C. H. , Lagadic‐Gossmann, D. , & Vaughan‐Jones, R. D. (1999). Characterization of intracellular pH regulation in the guinea‐pig ventricular myocyte. Journal of Physiology, 517(1), 159–180. 10226157 10.1111/j.1469-7793.1999.0159z.x PMC2269328

[tjp15085-bib-0006] Ma, J. , Gao, X. , Li, Y. , DeCoursey, T. E. , Shull, G. E. , & Wang, H.‐S. (2022). The HVCN1 voltage‐gated proton channel contributes to pH regulation in canine ventricular myocytes. The Journal of Physiology, 600(9), 2089–2103. 35244217 10.1113/JP282126 PMC9058222

[tjp15085-bib-0004] Satoh, H. , Delbridge, L. M. , Blatter, L. A. , & Bers, D. M. (1996). Surface:volume relationship in cardiac myocytes studied with confocal microscopy and membrane capacitance measurements: Species‐dependence and developmental effects. Biophysical Journal, 70(3), 1494–1504. 8785306 10.1016/S0006-3495(96)79711-4 PMC1225076

[tjp15085-bib-0005] Vinnakota, K. C. , & Bassingthwaighte, J. B. (2004). Myocardial density and composition: A basis for calculating intracellular metabolite concentrations. American Journal of Physiology Heart and Circulatory Physiology, 286(5), H1742–H1749. 14693681 10.1152/ajpheart.00478.2003

